# Supplementary figures and images for: ML-ROM wall shear stress prediction in patient-specific vascular pathologies under a limited clinical training data regime
Source: PLoS One. 2025 Jun 12;20(6):e0325644. doi: 10.1371/journal.pone.0325644 (PMC12161591; doi:10.1371/journal.pone.0325644)

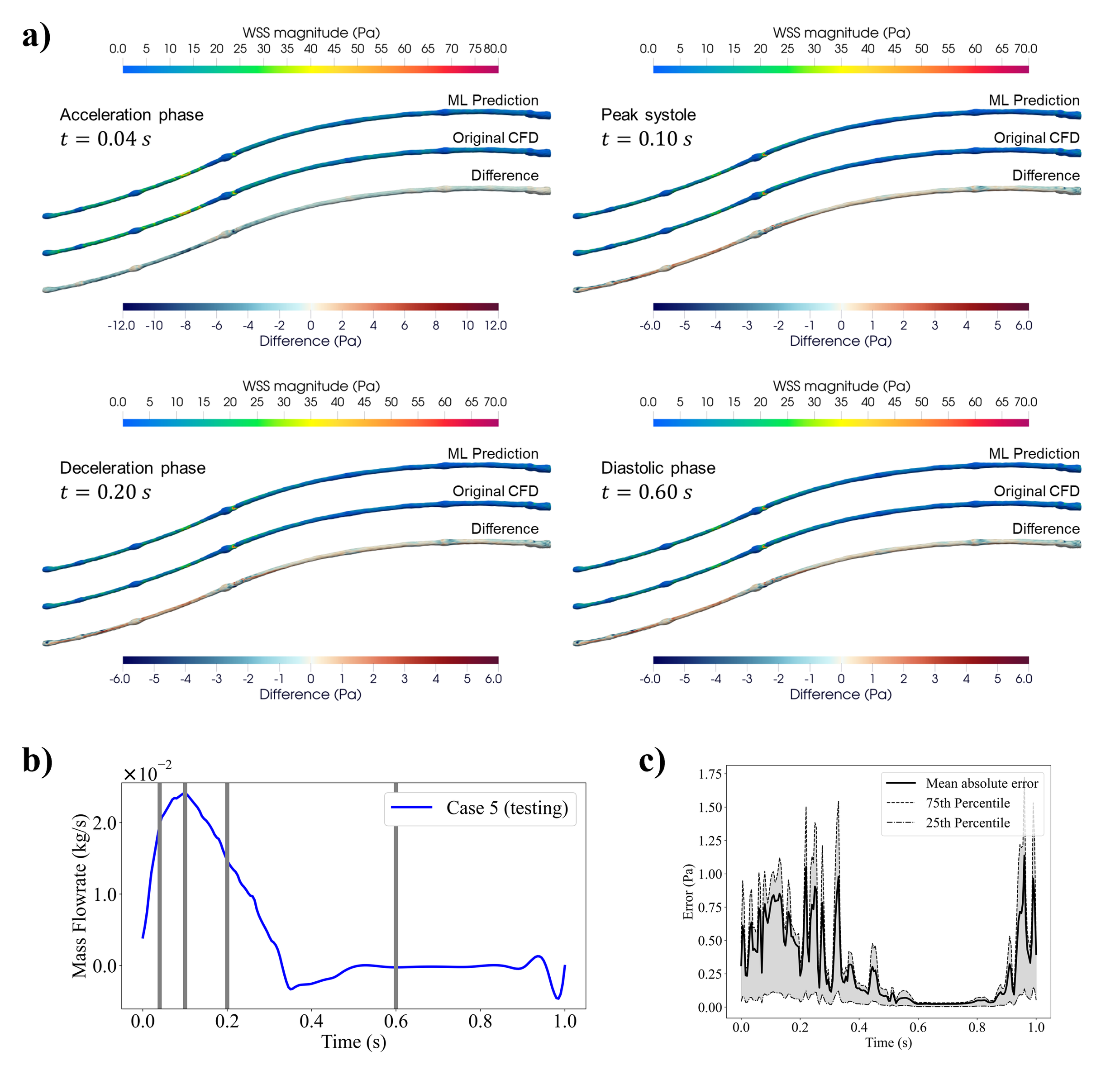

Supplement: S1 Fig — (a) 3D reconstructions of WSS in the PAD under μ5 (test case) at four states of the cardiac cycle: Acceleration, peak systole, deceleration, and diastole shown in (b). (c) Mean absolute error from the prediction of WSS over a cardiac cycle. Gray area shows the range between 75th and 25th percentile of error.). (TIFF) [file pone.0325644.s001.tif]

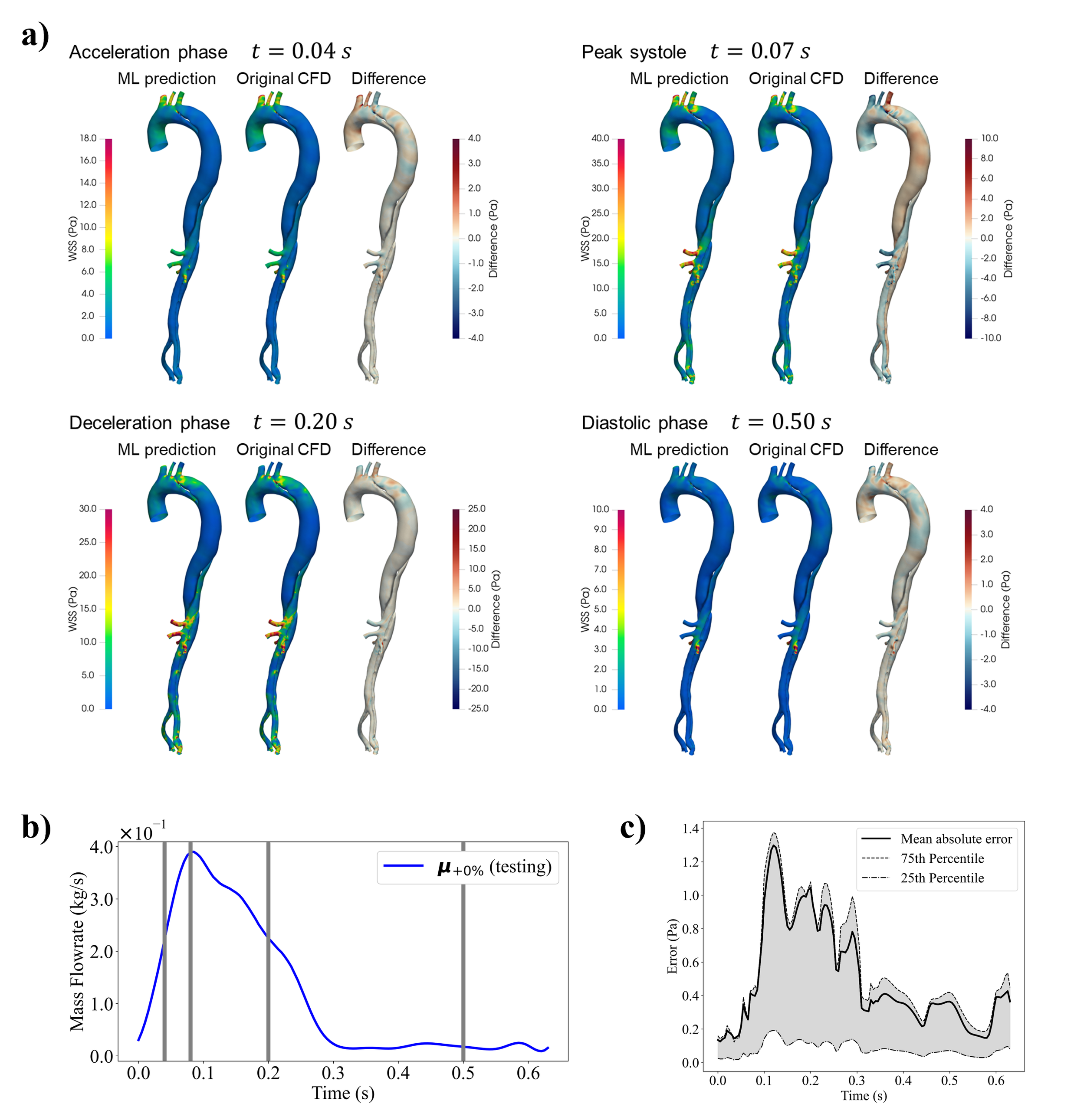

Supplement: S2 Fig — (a) 3D reconstructions of WSS in the AD under μ+0% (test case) at four states of the cardiac cycle: Acceleration, peak systole, deceleration, and diastole shown in (b). (c) Mean absolute error from the prediction of WSS over a cardiac cycle. Gray area shows the range between 75th and 25th percentile of error. (TIFF) [file pone.0325644.s002.tif]
